# The Constituent Phases and Micromechanical Properties of Steel Corrosion Layers Generated by Hyperbaric-Oxygen Accelerated Corrosion Test

**DOI:** 10.3390/ma16134521

**Published:** 2023-06-22

**Authors:** Baozhen Jiang, Kotaro Doi, Koichi Tsuchiya

**Affiliations:** 1 School of Materials, Sun Yat-sen University, Shenzhen 518107, China; 2 Research Center for Structural Materials, National Institute for Materials Science, Tsukuba 305-0047, Japan; doi.kotaro@nims.go.jp

**Keywords:** accelerated corrosion, steel reinforced concrete, corrosion products, microstructure, mechanical properties, nano-indentation

## Abstract

Hyperbaric oxygen-accelerated corrosion testing (HOACT) is a newly developed method to study in the labor the corrosion behavior of steel bars in concrete. This work aimed to intensively investigate the mechanical properties and microstructures of HOACT-generated corrosion products by means of nano-indentation tests, Raman micro-spectrometry, and scanning electron microscopy. The local elastic modulus and nanohardness varied over wide ranges of 6.8–75.2 GPa and 0.38–4.44 GPa, respectively. Goethite, lepidocrocite, maghemite, magnetite, and akageneite phases were identified in the corrosion products. Most regions of the rust layer were composed of a complex and heterogeneous mix of different phases, while some regions were composed of maghemite or akageneite only. The relationship between the micromechanical properties and typical microstructural features is finally discussed at the micro-scale level. It was found that the porosity of corrosion products can significantly influence their micromechanical properties.

## 1. Introduction

Reinforced concrete structures have been widely used all over the world. Outstanding properties and fair cost can be achieved by the coupling of steel with concrete. During the service life of reinforced concrete structures, the corrosion of steel bars in concrete is unavoidable. Moreover, it has been thought to be the most critical factor leading to the deterioration of reinforced concrete structures [1,2,3]. A very complex composition, consisting of a mixture of ferric oxyhydroxides and iron oxides, can be found in the resulting corrosion products, which form at the steel/concrete interface. Compared with the steel lost, the specific volumes of different types of corrosion products are 2–7 times larger [4,5]. This type of expansional stress caused by corrosion products can result in crack initiation, propagation, and even spalling of concrete cover.

**Figure 1:**
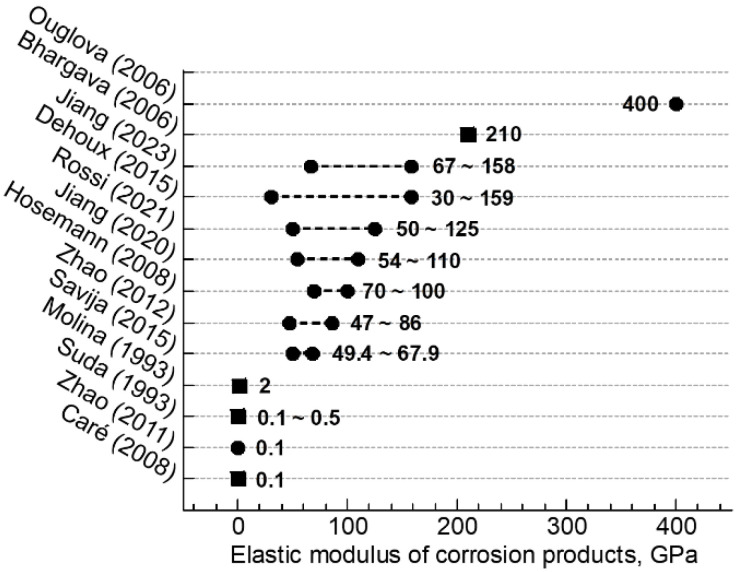
Elastic modulus of corrosion products reported in the literature [7,8,9,10,11,12,13,14,15,16,17,19,20]. Estimated and/or modeled values are indicated by squares, and experimentally measured values are indicated by circles.

Corrosion products play roles in the mechanical interactions between rebar and concrete [6]. Therefore, the mechanical properties of corrosion products, especially the elastic modulus, are essential parameters for modeling the cracking process. According to previous studies, which are summarized in Figure 1, the elastic moduli reported cover several orders of magnitude for corrosion products [7,8,9,10,11,12,13,14,15,16,17,18,19,20]. The first reason for such varying values consists of the different methods with which the elastic modulus was investigated. An elastic modulus obtained through an inverse analysis using finite element simulations is of the order of 0.1 GPa [7,8]. Such low input values are required in simulations for the purpose of obtaining realistic predictions of the cracking process of concrete cover. Zhao et al. [11] performed cyclic low-compression testing and oedometer testing of corrosion products, reporting that the elastic modulus was also 0.1 GPa. However, the corrosion products had been ground into small particles before testing, the measured elastic modulus of 0.1 GPa might be not real for bulk corrosion products. In contrast, as indicated by most of the solid circles in Figure 1, the elastic modulus obtained through direct experimental measurements is much higher. Since the corrosion product layer is usually very thin, depth sensing indentation is an effective experimental method for characterizing the elastic modulus of corrosion products [13,14,15,16,17,18,19,20]. The corrosion products investigated by indentation methods in previous studies can be divided into two types: those generated by anodically accelerated corrosion and those generated in nature. The former corrosion products exhibited an elastic modulus of 47 GPa on average in [14], 56 GPa on average in [19], 49.4–67.9 GPa in [13], and 54–110 GPa in [16]. For the latter corrosion products, the elastic modulus was 61–86 GPa in [14], 64–159 GPa in [19], 50–125 GPa in [17], and 67–158 GPa in [20]. The elastic moduli obtained by indentation tests are still quite different. It is believed that the corrosion products generated under different exposed environments and operating conditions should have different constituent phases and morphologies.

To understand the complex steel bar corrosion in reinforced concrete structures, accelerated corrosion test methods are usually performed in the lab. The conventional methods include the impressed current method and the wet–dry cycle method. However, there are some issues related to these two methods. For example, the corrosion products generated by the impressed current method present a higher expansion ratio than the natural corrosion products because this type of artificial corrosion product usually contains a large amount of Cl^−^, which is not found in nature [21]. The corrosion products generated by the wet–dry method are similar to those generated in nature. However, it takes a long time to achieve the corrosion of steel bar because it the inside of the concrete cover is slow to dry [22]. Recently, a new method called hyperbaric oxygen-accelerated corrosion testing (HOACT) was developed [23,24,25]. The oxygen reduction reaction on the steel surface can be promoted by sufficient oxygen. Meanwhile, the anodic reaction can be promoted by the Cl^−^ added to the concrete. This process relates to the breakdown of passive film and the dissolution of steel. The above two factors contribute to the rapid corrosion of steel in concrete. More importantly, the composition of corrosion products generated by HOACT is almost identical to that generated in the real environment. Therefore, it is believed that HOACT is a better method for studying reinforcing corrosion in concrete.

As a newly developed accelerated corrosion method, HOACT-induced steel corrosion products still lack a systematic and in-depth study. Therefore, the present study aimed to provide an intensive analysis of the micromechanical properties and microstructures of corrosion products generated by HOACT. A series of nano-indentation tests were performed to investigate the elastic modulus and nanohardness of local corrosion products. At the same time, the phase compositions and morphologies of these local corrosion products were investigated by Raman micro-spectrometry, energy dispersive X-ray spectrometry (EDS), and scanning electron microscopy (SEM). These observations help us to comprehensively understand the corrosion products at the micro-scale level and then to clarify the relationships between microstructural features and mechanical properties.

## 2. Materials and Methods

### 2.1. Specimen Preparation

A commercial steel bar of Japanese Industrial Standard (JIS) SD345, with a chemical composition of Fe-0.26C-0.19Si-0.88Mn-0.029P-0.018S (wt.%), was used in this study. This steel is equivalent to Grade 40 from American Society for Testing and Materials (ASTM) A615. The diameter of this steel bar is 19 mm. It was embedded in a cylindrical mortar block. This block was 30 mm in diameter, 30 mm in height, and 5.5 mm in cover thickness, and its upper and lower surfaces were sealed with epoxy resin. The weight ratio of 2.06 M NaCl solution cement: fine aggregates was 0.6:1:3 in the mortar. The mortar block was subjected to a curing process for 28 days at room temperature and at 95% relative humidity. Then, it was placed in a high-pressure container. The schematic illustration of the HOACT apparatus was reported by Doi et al. [25]. During HOACT, the internal oxygen pressure was kept at 0.6 MPa, the relative humidity was greater than 95%, and the dissolved oxygen concentration of the mortar block increased. This accelerated corrosion test was performed for 14 days (S1) and 56 days (S2), respectively. After HOACT, the specimen was embedded in cold mounting resin. Grinding (SiC abrasive papers of 280, 400, 600, and 1000 grit) and polishing (polishing suspensions with particle sizes of 9 µm, 6 µm, 3 µm, and 0.06 µm) were performed on an Ecomet 250 Pro Grinder-Polisher machine to produce a mirror surface. The block was cleaned with acetone for 20 min by an ultrasonic cleaner and then dried.

### 2.2. Nano-Indentation Tests

Nano-indentation tests were conducted to investigate the micromechanical properties of the corrosion product layer. The Hysitron Triboindenter TI950 with a Berkovich tip was used in the present study. Multiple different areas of the corrosion product layer were selected for nano-indentation tests, and the number indentation points in each area varied between 10 and 20. The indentations were separated from each other by a distance of 10 µm. The total number of indentation points made on S1 and S2 were 66 and 279, respectively. Nano-indentation tests were performed in a load control mode. The load increased at a rate of 250 µN/s to 10 mN, holding for 20 s at this force and then decreasing the load at a rate of 250 µN/s. Based on the Oliver and Pharr method [26], the elastic modulus and nanohardness were calculated from the obtained load-depth curves.

### 2.3. Microstructural Characterization

The phase compositions of corrosion products were identified by micro-Raman analysis (inVia Reflex, Renishaw). Raman measurements were performed at a laser power of 1% of the maximum and at an excitation wavelength of 532 nm. The spectra were recorded using a 50× objective lens with an acquisition time of 5 min. The acquisition and treatment of the spectra were performed with WiRE 5 software. These obtained Raman spectra were analyzed by comparing them with the reference spectra previously reported in the literature [27,28,29]. SEM observations in backscatter electron (BSE) mode and EDS analysis were conducted at 20 kV with an FEI Quanta FEG 250. The composition of each indentation was quantitatively investigated by EDS spot analysis.

## 3. Results and Discussion

### 3.1. Microstructural Characterization of Corrosion Products

**Figure 2:**
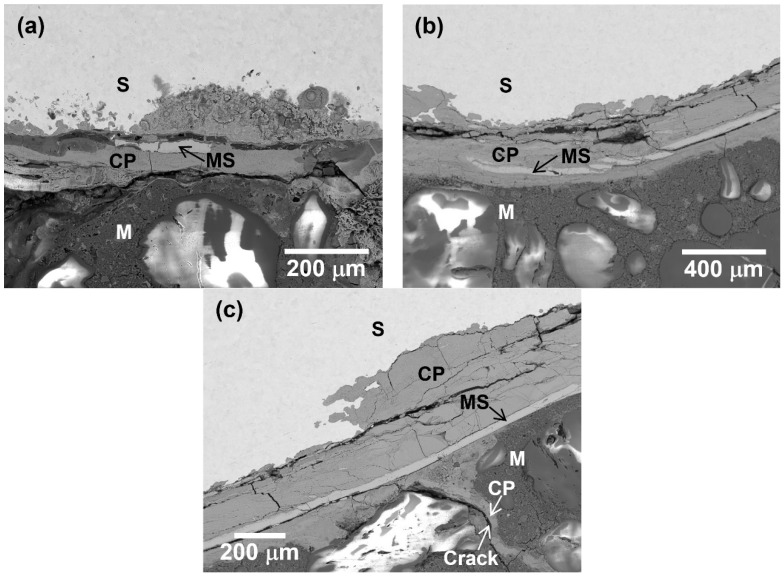
SEM-BSE micrograph of corrosion products generated by HOACT: (**a**) S1; and (**b**) and (**c**) S2. S: steel; M: mortar; CP: corrosion products; MS: mill scale.

Figure 2 shows the BSE micrographs of corrosion products at the steel/mortar interface. The thickness of the corrosion product layer was approximately 55 µm for S1 and 350 µm for S2. There were many cracks with different sizes in the corrosion product layer. Some cracks were parallel to the corrosion product layer, while some cracks were perpendicular to the corrosion product layer. A bright layer with a thickness of around 30 µm could be found inside the corrosion products, as indicated by the black arrows. It was the original mill scale that was not generated during HOACT process but that formed on the steel surface during the manufacturing process, such as hot rolling or forging [20,25]. The mill scale contained some initial defects, including cracks, exfoliation, and so on. It has been reported that crevice corrosion between the steel substrate and the mill scale is induced by Cl^−^, which can intrude through these initial defects in the mill scale [25,30]. With the gradual generation of corrosion products, the resulting volume expansion can break down the mill scale. The mill scale is visible near to or far from the steel surface, depending on how much the corrosion has propagated. In addition, as shown in Figure 2c, the volume expansion caused by corrosion products also initiated cracks in the mortar, and some corrosion products penetrated into the mortar along cracks. The corrosion of steel bar will progress more rapidly after cracking in the mortar because the cracks can provide a fast-transportation path for oxygen and Cl^−^. 

**Figure 3:**
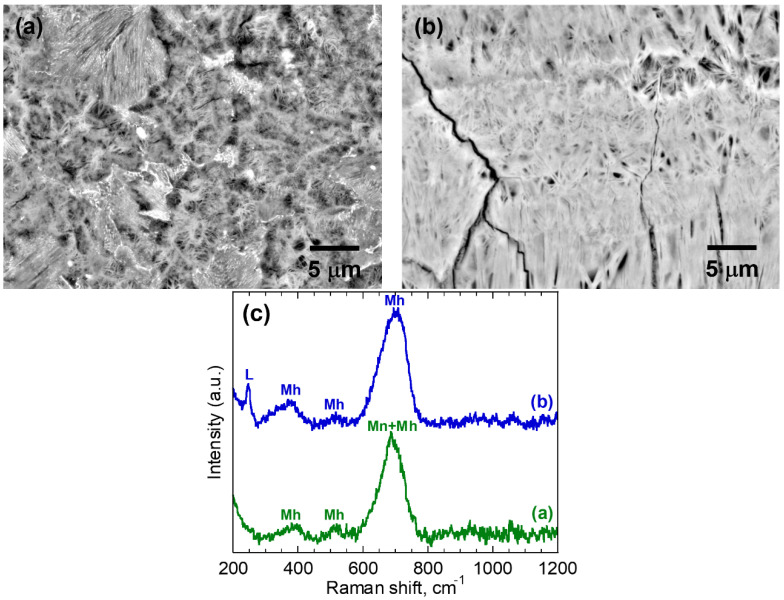
Microstructural characterization of the corrosion products in S1: (**a**,**b**) BSE-SEM micrograph; and (**c**) corresponding Raman spectra. L: lepidocrocite; Mh: maghemite and Mn: magnetite.

Based on the micro-Raman analysis, the phases identified in S1 included lepidocrocite (γ-FeOOH), maghemite (γ-Fe_2_O_3_), and magnetite (Fe_3_O_4_). The detailed morphologies of corrosion products in S1 and corresponding Raman spectra are shown in Figure 3. These two BSE images show that the corrosion products were loose and porous. For the Raman spectra shown in Figure 3c, the presence of the sharp peak at 702 cm^−1^ can be attributed to maghemite, while the presence of the peak at 687 cm^−1^ is due to a combination of scattering intensities from magnetite and maghemite. Similar results have been previously discussed in the literature [27]. Therefore, the microstructure shown in Figure 3a consists of a mix of maghemite and magnetite. However, it is difficult to distinguish magnetite from maghemite in this BSE image. Figure 3b shows a microstructure consisting of a mix of maghemite and lepidocrocite. The needle-shaped crystals in the corrosion products are lepidocrocite phase, which usually forms in the early period of exposure and is unstable [31,32,33,34].

**Figure 4:**
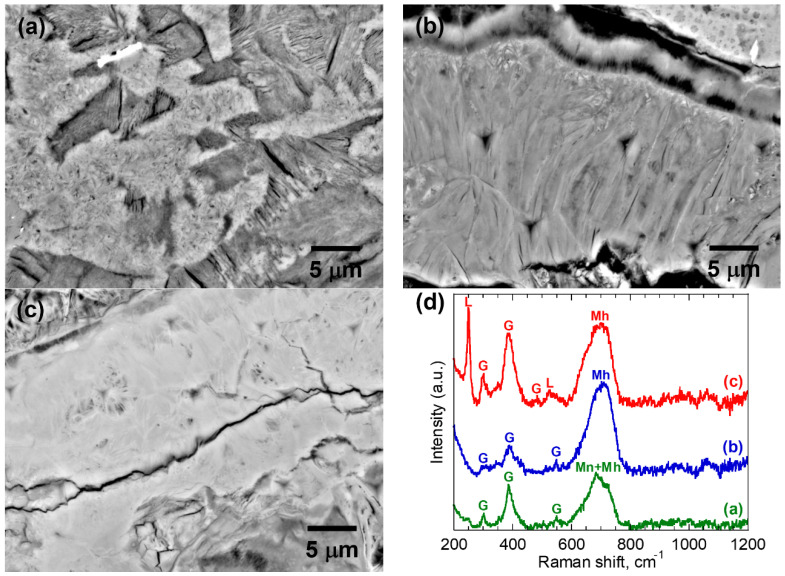
Microstructural characterization of the corrosion products in S2: (**a**–**c**) BSE-SEM micrograph; and (**d**) corresponding Raman spectra. L: lepidocrocite; G: goethite; Mh: maghemite and Mn: magnetite.

The corrosion products in S2 presented more diverse morphologies. Most regions of the corrosion products in S2 contained a mixture of several phases. In addition to the three phases identified in S1, the stable goethite (α-FeOOH) phase was also found in S2. Figure 4 shows three representative BSE morphologies and the corresponding Raman spectra. Maghemite and goethite were identified in all these microstructures. In addition, the microstructure shown in Figure 4a,c also contains magnetite and lepidocrocite, respectively. 

**Figure 5:**
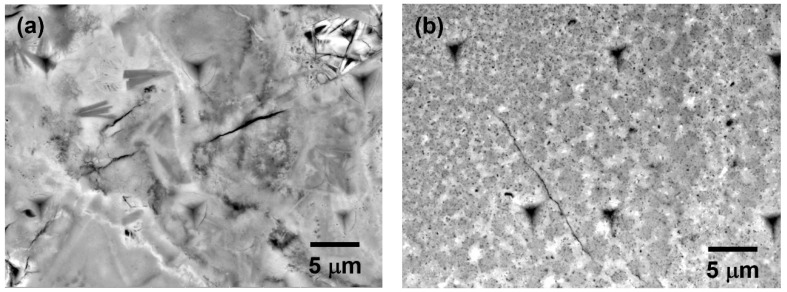

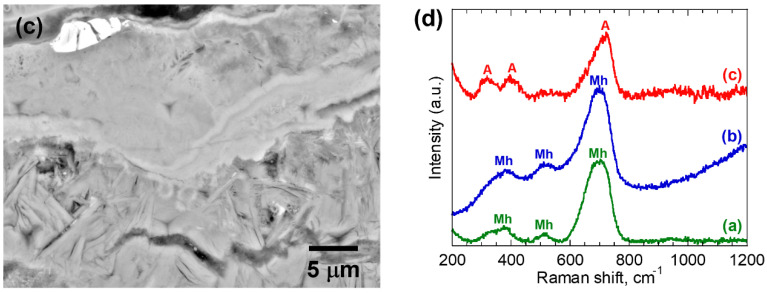
Microstructural characterization of the corrosion products in S2: (**a**–**c**) BSE-SEM micrograph and (**d**) corresponding Raman spectra. A: akageneite and Mh: maghemite.

Moreover, some local areas of the corrosion products in S2 contained a single phase. Figure 5a exhibits the BSE morphology containing only akageneite (β-FeOOH). Akageneite is an iron oxyhydroxide mineral with a tunnel structure stabilized by inclusion of chloride. The NaCl solution mixed in the concrete contributed to the generation of the akageneite phase. Both BSE morphologies shown in Figure 5b,c contain only maghemite. However, it is clear that these two morphologies are totally different. Figure 5c presents a more compact and denser microstructure compared with Figure 5b.

### 3.2. Characterization of the Mechanical Properties

**Figure 6:**
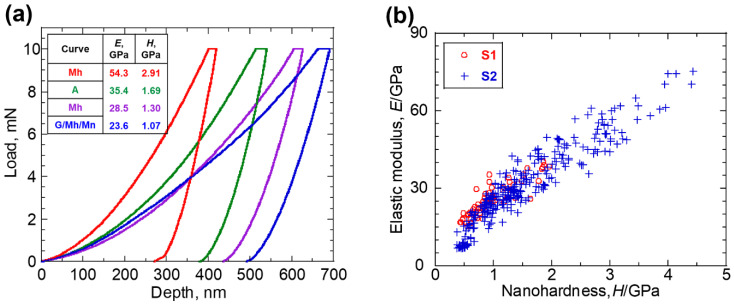
(**a**) Typical nano-indentation load-depth curves obtained from different corrosion products; and (**b**) elastic modulus E and nanohardness H measured on all the indentations.

Figure 6a shows some typical nano-indentation load-depth curves obtained from different corrosion products. The inserted table lists the elastic modulus E and nanohardness H calculated from corresponding load-depth curve. A steeper curve in the unloading part suggests a higher elastic modulus, and a larger residual indentation depth suggests a lower hardness. The nano-indentation results for all the investigated corrosion products are plotted in Figure 6b. Clearly, a rising trend in the elastic modulus can be seen as the nanohardness increases. The number of indentation points made on S1 was 66 points. The elastic modulus was between 14.5 and 40.5 GPa (25.9 ± 6.4 GPa on average), while nanohardness was between 0.43 and 1.96 GPa (0.98 ± 0.40 GPa on average). The number of indentation points made on S2 was 279 points. Nano-indentation investigations covered more various microstructures, leading to a much wider range in the values of elastic modulus and nanohardness. The elastic modulus varied between 6.8 and 75.2 GPa (31.5 ± 14.7 GPa on average), while nanohardness varied between 0.38 and 4.44 GPa (1.55 ± 0.88 GPa on average).

### 3.3. Relationship between Local Microstructures and Mechanical Properties

As mentioned above, the HOACT generated corrosion products consisting of several phases, presenting a complex and heterogeneous microstructure and a wide range of mechanical properties. The mechanical properties per phase should be different. Although the related studies have been limited, the elastic modulus and nanohardness of each phase showed an ascending order of goethite < maghemite < magnetite [16,19,20]. For the microstructures mixing with various phases, there is a rough tendency that the elastic modulus and nanohardness become greater by increasing the Fe/O atomic ratio, although the values at certain Fe/O atomic ratios still show a broad range [16]. However, this study showed inconsistent results. Table 1 lists the mechanical properties corresponding to the microstructures shown in Figure 3, Figure 4 and Figure 5. It is clear that there was no significant, direct correlation between the mechanical properties and the phase compositions (or the Fe/O atomic ratio) for the studied corrosion products.

By examining the morphologies of corrosion products, it is believed that the microstructural porosity greatly influences the mechanical properties of corrosion products. Based on these BSE images, the porosity of some corrosion products was measured using the ImageJ software package (v1.8.0.112). For the microstructures composed of several phases, the microstructures shown in Figure 3a,b and Figure 4a have high porosity, which is approximate 17.5%, 14.5%, and 12.7%, respectively. The corresponding elastic modulus and nanohardness listed in Table 1 are quite low. Meanwhile, a more compact and denser microstructure shown in Figure 4b,c has a higher elastic modulus and nanohardness. This kind of phenomenon can also be found in the microstructures composed of single maghemite phase. The more porous microstructure shown in Figure 5b, which has a porosity of about 7.4%, has a lower elastic modulus and nanohardness than the microstructure shown in Figure 5c.

**Figure 7:**
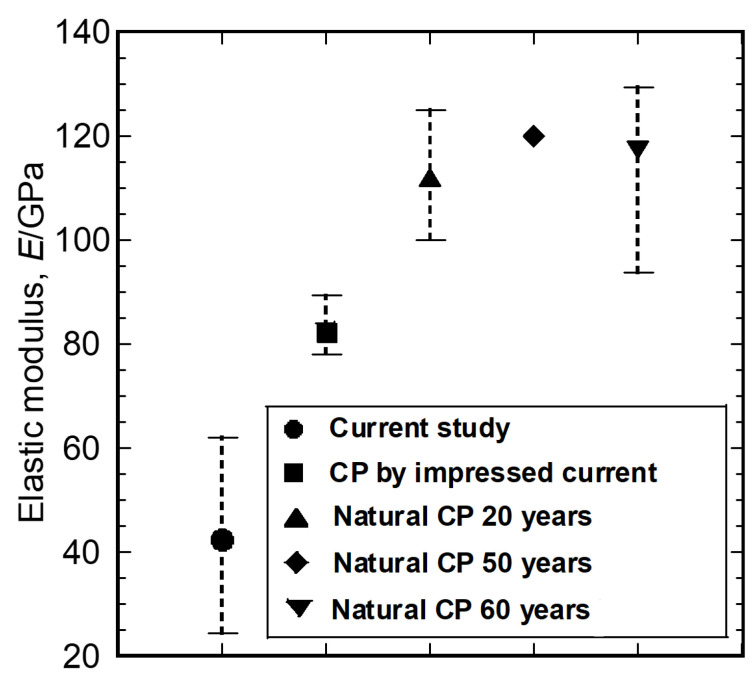
Elastic modulus of maghemite phase reported in previous studies and the present study [16,17,19,20]. The whiskers show the maximum and minimum values, while the solid symbols indicate the average values. CP: corrosion products.

The elastic moduli for maghemite phase reported by previous studies [16,17,19,20], as well as by the current study, are summarized in Figure 7. The same phase investigated in different studies exhibited different elastic moduli, and a much lower elastic modulus is reported in the current study. The corrosion products generated under different environments should have different porosities. Gao et al. [35] reported that a higher corrosion rate resulted in higher porosity. The natural corrosion process is slow, so the corrosion products would densely accumulate at the interface between the steel bar and the cement. The accelerated corrosion test method results in a rapid formation of corrosion products and consequent loose morphologies. This fact can explain why the elastic modulus and nanohardness reported in the literature were higher for natural corrosion products than for artificial corrosion products. Another noteworthy phenomenon is that the porosity of the corrosion product layer likely decreases over time as its thickness increases [35,36,37,38]. The much thinner corrosion product layer obtained in the present study is more porous and looser than those reported by previous researchers, exhibiting a much lower elastic modulus and nanohardness.

In addition, some researchers have speculated that the mechanical properties might also be affected by some other microstructural parameters, one of which is the crystallinity of corrosion products [19,20]. Unfortunately, this issue cannot be assessed by the present investigation. Based on the previous studies, as well as the present study, the characteristic mechanical properties of corrosion products should not be regarded as constants. To predict accurate values of elastic modulus and nanohardness, many microstructural parameters intrinsic to corrosion products, such as phase composition, porosity, crystallinity, and so on, should be considered.

## 4. Conclusions

The corrosion of steel bar in concrete was accomplished using a newly developed accelerated corrosion method, HOACT. The nano-indentation method was applied to explore the micromechanical properties of the corrosion products. SEM and micro-Raman analysis were conducted to characterize the microstructures of the corrosion products. The following conclusions were drawn.

1. The phases identified in the corrosion products included goethite, lepidocrocite, maghemite, magnetite, and akageneite, presenting themselves as mixtures in most regions of the corrosion product layer at the microscopic level. Some local regions were composed of a single maghemite or akageneite phrase were also found.
2. The micromechanical properties of HOACT-generated corrosion products covered a wide range. The elastic modulus varied between 6.8 and 75.2 GPa, while the nanohardness varied between 0.38 and 4.44 GPa.
3. The microstructural porosity greatly influenced the micromechanical properties of the corrosion products. A more porous and looser microstructure usually corresponds to a lower elastic modulus and nanohardness.
4. According to the previous studies, as well as the current study of corrosion products, the characteristic mechanical properties per phase should not be considered a constant value. They can change with corrosion conditions.

## Figures and Tables

**Table 1:** Composition and average values of elastic modulus E and nanohardness H, corresponding to the microstructures shown in Figure 3, Figure 4 and Figure 5. L: lepidocrocite; G: goethite; A: akaganeite; Mh: maghemite and Mn: magnetite.

Figure		Fe/O Atomic Ratio	Phases	*E* (GPa)	*H* (GPa)
3	(a)	0.66 ± 0.06	Mh/Mn	22.6 ± 4.5	0.79 ± 0.25
	(b)	0.60 ± 0.02	L/Mh	31.5 ± 5.2	1.27 ± 0.40
4	(a)	0.65 ± 0.02	G/Mh/Mn	23.5 ± 3.4	1.02 ± 0.27
	(b)	0.62 ± 0.01	G/Mh	40.2 ± 3.0	2.14 ± 0.29
	(c)	0.57 ± 0.02	L/G/Mh	51.8 ± 5.5	3.18 ± 0.41
5	(a)	0.51 ± 0.02	A	35.4 ± 3.3	1.55 ± 0.27
	(b)	0.65 ± 0.01	Mh	28.3 ± 1.8	1.32 ± 0.07
	(c)	0.65 ± 0.02	Mh	54.6 ± 5.2	2.79 ± 0.42

## Data Availability

The raw/processed data required to reproduce these findings cannot be shared online at this time due to technical limitations, but they are available by contacting the corresponding author.
